# Research on the Influence of Cold Drawing and Aging Heat Treatment on the Structure and Mechanical Properties of GH3625 Alloy

**DOI:** 10.3390/ma17112754

**Published:** 2024-06-05

**Authors:** Ji Li, Yujie Wo, Zhigang Wang, Wenhao Ren, Wei Zhang, Jie Zhang, Yang Zhou

**Affiliations:** 1 School of Mechanical Engineering, Zhejiang University of Technology, Hangzhou 310014, China; 2 Hubei Key Laboratory of High-Quality Special Steel, Daye Special Steel Co., Ltd., Huangshi 435001, China; 3 Research Institute for Special Steel, Central Iron and Steel Research Institute, Beijing 100081, China; 4 CITIC Metal Co., Ltd., Beijing 100004, China

**Keywords:** GH3625 alloy, cold drawing, mechanical properties, dislocation strengthening

## Abstract

With the development of the petroleum industry, the demand for materials for oilfield equipment is becoming increasingly stringent. The strength increase brought about by time strengthening is limited in meeting the needs of equipment development. The GH3625 alloy with different strength levels can be obtained through cold deformation and heat treatment processes. A study should be carried out to further develop the potential mechanical properties of GH3625. In this study, the GH3625 alloy was cold drawn with different reductions in area (0–30%) and heat treated, and its mechanical properties were tested. The microstructure of the alloy during deformation and heat treatment was characterized by methods such as optical microscopy (OM), scanning electron microscopy (SEM), and transmission electron microscopy (TEM) based on the principles of physical metallurgy. The strength increase caused by dislocation strengthening was calculated from the dislocation density, tested by X-ray diffraction (XRD). The calculated value was compared to the measured value, elucidating the strengthening effect of cold deformation and heat treatment. The results showed that the yield strength and yield ratio of the cold-drawn alloy significantly reduced after aging at 650 °C and 760 °C. Heat treatment can make a cold-deformed material recover, ablate dislocations, and greatly reduce the dislocation density in the microstructure of the GH3625 alloy, which was the main factor in the decrease in yield strength. The work-hardening gradient of the cold-drawn material varied greatly with different reductions in area. When the reduction in area was small (10%), the hardness gradient was obvious. When it increased to 30%, the alloy was uniformly strengthened as the deformation was transmitted to the axis. This study can provide more mechanical performance options for GH3625 alloy structural components in the petrochemical industry.

## 1. Introduction

The GH3625 superalloy is a solid-solution-strengthened nickel-based deformation superalloy, with Mo and Nb as the main strengthening elements. With excellent corrosion resistance and oxidation resistance, it has good tensile and fatigue properties from low temperature to 980 °C, as well as resistance to salt spray corrosion. GH3625 is often used in the chemical processing, power, aerospace, and automobile industries owing to its unique combination of high strength, excellent fabricability and weldability, and outstanding corrosion resistance [1,2,3,4].

With the development of the petroleum industry in recent years, the service conditions of materials have become increasingly rigorous. As the depth of oil wells increases, oil extraction equipment is exposed to higher temperatures and pressures, as well as acidic gas. GH3625 superalloy bars produced by the cold drawing process are commonly made into piston rods for oil drilling [5,6,7]. The original intention of the GH3625 material’s design was to have excellent corrosion resistance, while mechanical properties were not its main advantage. However, for structural materials, higher strength and more alternative strength grades can significantly expand the application range. The strength of conventional GH3625 can be appropriately enhanced through aginge strengthening. Because the effect of the second phase is limited by its chemical composition, the mechanical properties of the material can be significantly improved through cold deformation from another dimension. Cold deformation has both advantages and disadvantages. Its advantages are its high processing efficiency and obvious strengthening effect. However, its disadvantages are that the deformed steel bar has a great residual stress that cannot be removed, and is prone to deformation as the stress is released during service. Aging treatment after cold drawing may be a good solution. After a large number of dislocations are generated after cold drawing, the residual stress can be appropriately released by heat treatment. Although some dislocations may ablate at this time, the second phase precipitated during aging can compensate for the lost strength. Therefore, elucidating the balance between the size of deformation and the aging treatment process based on the principles of physical metallurgy is the key to achieving microstructure and performance control. However, researchers have paid more attention to welding and hot working for the GH3625 superalloy and similar alloys, compared to the effects of cold drawing deformation and subsequent heat treatment on the microstructure and properties [8,9,10,11,12,13,14,15].

Cold deformation can not only change the shape and size of the material, but also alter the microstructure, thereby affecting its properties. Ding studied the influence of cold drawing with different reductions in area on the location of δ-phase precipitation, indicating that when the strain was 35% the δ phase first nucleated and precipitated at grain boundaries and deformation twin boundaries, and then, nucleated and grew within the grain. When the strain was ≥50%, the δ phase first nucleated and grew at the deformation twin boundaries, grain boundaries, and deformation bands, and then, nucleated and precipitated within the grains. And as the strain increased, the δ phase precipitated more in the deformation bands [16,17,18]. Zhao et al. studied the effect of different cold deformation on the mechanical properties, it was found that an increase in the cold deformation rate could result in more cold work hardening, leading to a significant improvement in tensile strength and a decrease in the elongation of the alloy at room temperature [19]. Wang Zhigang et al. studied the effects of different reductions in area and deformation passes on the microstructure and properties of alloys. It was found that the grains were elongated during cold deformation, resulting in deformation structures such as dislocation cells and deformation twins, which increased the resistance of dislocation movement and led to work hardening. Cold drawing was a gradual diffuse process from the outside to the inside. When the reduction in area varied between 19% and 32%, as the reduction in area increased, the hardness of the alloy steadily, but not significantly, increased [20,21]. The relationship between the performance at room temperature and the reduction in performance of the cold-drawn GH3625 alloy was also revealed. However, the yield strength ratio of the produced bars was up to 0.99 or above, greatly reducing their safety.

A few researchers have studied the effect of heat treatment after cold deformation on alloy properties. Qin et al. studied the effects of different heat treatment temperatures on the microstructure, mechanical properties, and intergranular corrosion resistance of the GH3625 alloy after cold deformation. It was found that as the heat treatment temperature increased, grains engulfed each other, grain boundaries moved, and grains grew. The amount of the precipitated phases in the matrix gradually decreased with increasing temperature, but some precipitated phases increased [22].

Gao Yubi et al. studied the microstructure evolution of cold-deformed GH3625 alloy pipes during the intermediate annealing process. It was found that with the degree of cold deformation increasing, the uniformity of the alloy microstructure gradually improved, and the hardness value significantly increased, especially at cold deformation degrees from 0 to 50%. It was also found that at an annealing temperature of 1120 °C, complete recrystallization occurred and the recrystallized grain size decreased as the cold deformation rate increased. As above, the research data on the mechanical properties of the GH625 alloy after cold drawing and heat treatment with different reductions in area is not sufficient. Therefore, it is necessary to systematically study the microstructure and mechanical properties of the GH3625 alloy after cold drawing and aging treatment [23,24].

In this paper, the microstructure and mechanical properties of GH3625 alloy bars in different conditions were studied by adjusting the reduction in area and aging temperatures, in order to optimize their microstructure and performance. Meanwhile, the study can also lay the basis for further research on other high-temperature alloys, provide solutions for selecting GH3625 alloys with different mechanical indicators in the subsequent equipment design process, and provide experimental data and a theoretical basis for designing and manufacturing more advanced and safer oil and gas equipment. The process provided by this research can also improve the problem of residual stress causing bending of cold-drawn parts during service.

## 2. Material and Experimental Procedure

For this study, GH3625 ingots of Φ360 mm were built with vacuum induction melting (VIM) + vacuum arc remelting (VAR), using niobium chromium master alloys (the chemical composition is shown in Table 1, andsupplied by CITIC Metal Co., Ltd., Beijing, China), nickel plates (≥99.96 wt%), metallic molybdenum (≥99.85 wt%), etc. as raw materials. The chemical composition of GH3625 was measured by a fluorescence spectrometer and calibrated with a GH3625 standard sample. The chemical composition of the alloy is shown in Table 2.

The GH3625 ingot was held at 1150 °C in a natural gas heating furnace, and formed into square steel billets of 100 mm × 100 mm by a pneumatic hammer. The billets were forged and rolled into bars of Φ20 mm. The bars were annealed at 980 °C for 1 h, followed by air cooling and polishing into bars of Φ18 mm, which were acidized and saponified, and cold-drawn in a 20-ton double-chain cold drawing machine with different reductions in area (10%, 20%, 30%) at a speed of 10.32 m/min. (Deformation at room temperature is called cold deformation or cold drawing in this paper). The 12 cold-drawn bars were divided into 3 groups, with each group consisting of 4 bars. The first group was kept in the cold-drawn state, and the other two groups were aged in resistance furnaces. One group was held at 760 °C in air for 1 h, followed by natural air cooling. The other group was held at 650 °C in air for 24 h, followed by natural air cooling. These three groups of bars were tensile tested once per experiment, with an error range of no more than ± 1%. The hardness of the samples before cold drawing, after cold drawing without heat treatment, and after heat treatment was determined by an EV500-2A semi-automatic Vickers hardness tester with a test loading of 10 kg. Three pieces of 3 mm thick sheets were taken parallel to their cross-section from three different state bars for the experiments, followed by statistical analysis of their mean and variance. Points at intervals of 0.5 mm from the center to the edge on the cross-section of the samples were tested to analyze the hardness distribution. The tensile performance and hardness at room temperature were measured and the grain structure, precipitated phase, and dislocation density of the samples were analyzed.

The tensile performance includes tensile strength, yield strength, elongation, and section shrinkage. The sampling position was at the center of the bar in the axial direction. The samples for tensile testing were uniformly processed according to the specified proportion of samples with an available part diameter of 5 mm and a gauge distance of 25 mm. The tensile test was carried out by a Zwick-Z400-type tensile testing machine at room temperature. The tensile rate within the elastic deformation range was 3 mm/min, which increased to 10 mm/min when the stress exceeded the yield strength.

A 20 mm steel rod was selected and dissected along the central axis, and polished with abrasive paper. And then, it was etched using a mixture of copper chloride and hydrochloric acid for characterization by OM and SEM. The metallographic structure was observed using a Zeiss 40MAT optical metallographic microscope with a magnification of 500 times. The microstructure was characterized using a FEI-Quanta650 FEG thermal field emission scanning electron microscope at a magnification of 4000 times, with a working distance of 16–18 mm and an electron acceleration voltage of 20 kV. A 0.3 mm thick slice was cut from the cross-section of the bar by wire electric discharge machining, and then, polished to 50 µm with #1000 abrasive paper, and cut into a wafer of Φ3 mm. A mixed solution of HClO_4_ and C_2_H_5_OH was used for electrolytic double spraying to make a TEM sample with an area of a certain thinckness.

At the same time, TEM (FEl Tecnai G2 F20) operating at a voltage of 200 kV was utilized to observe the microstructure of samples, and a Bruker energy spectrometer was equipped to semi-quantitatively characterize the composition. An X-ray diffraction test was performed using a German Brooke D8 ADVANCE X-ray diffractometer, with the following process parameters: Co target with wave length of 1.7889 Å, tube current of 40 mA, tube voltage of 35 kV, scanning speed of 2°/min, and Lynxeye XE detector.

## 3. Results and Discussion

### 3.1. Microstructure

**Figure 1:**
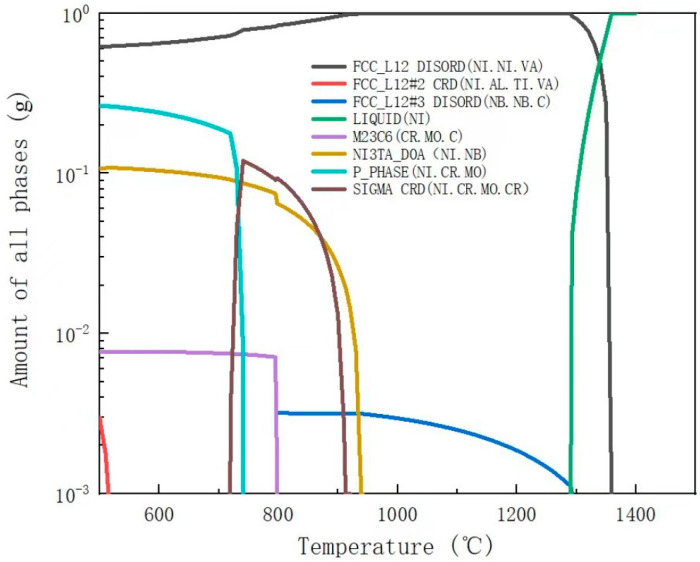
Equilibrium content of phases in GH3625 alloy calculated by Thermal-Calc.

Thermal-Calc (TCNI10: Ni-Alloys v10.0) was used to analyze the microstructure of the GH3625 alloy in a complete equilibrium state. The composition system used in the calculation is shown in Table 1, with a temperature range of 500–1500 °C and a total amount of 1 g. The thermodynamic calculation results are shown in Figure 1.

**Figure 2:**
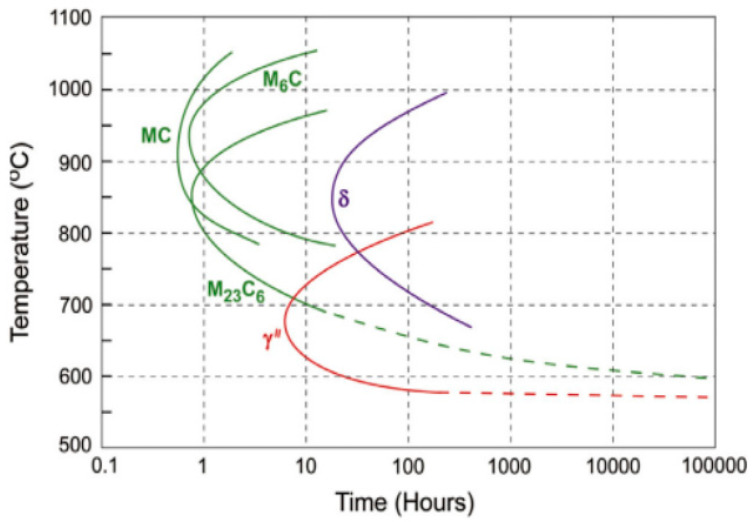
TTT of GH3625 alloy [25,26,27].

Florren S drew a time–temperature–transformation (TTT) plot of the GH3625 alloy, as shown in Figure 2.

The microstructure of the GH3625 alloy is analyzed based on Figure 1 and Figure 2. Figure 1 shows that the GH3625 matrix is austenite. In a fully equilibrium state, if the temperature of the alloy liquid drops to 1355 °C, the austenite begins to form, and the liquid phase completely solidifies at 1295 °C. When the temperature is above 940 °C, there is a small amount of MX phases which have a face-centered cubic (FCC) structure, mainly composed of niobium carbide (NbC), with a mass fraction not exceeding 0.3% (shown as the blue line in Figure 1, FCC-L12 # 3). The MX phase is commonly referred to as primary carbide. Residual carbide and niobium distributed on the interdendritic zone combine in the early stages of solidification. If there are nitride and oxide inclusions in the liquid phase, it will further promote the nucleation of NbC. However, the NbC phase is unstable at temperatures of 700–950 °C and easily decomposes into the M23C6 or M6C phase, while the NbC phase could also decompose into the M6C phase at temperatures of 800–980 °C. Some researchers believe that the M6C phase is transformed from M23C6, and the final form is M6C [28], while the author believes that the M6C phase and M23C6 may coexist, and their content ratio depends on the aging temperature and the Cr/Mo ratio of the material itself. When the temperature is less than 800 °C, 0.08 wt% M23C6 phase always exists in a thermodynamic equilibrium (shown as the purple line in Figure 1) [29].

Except for carbides, the main precipitates in the microstructure are the δ phase and the γ″ phase. When the temperature drops to 940 °C, the δ phase forms in a thermodynamic equilibrium state, composed of Ni_3_Nb, with a maximum content of 10 wt% (shown as the brown line in Figure 1, NI3TA). Because the γ″ phase is the metastable phase of the δ phase, the γ″ phase does not exist in the thermodynamic equilibrium state. The δ phase has an orthorhombic structure (D0a), and the γ″ phase has an ordered body-centered tetragonal structure (D022). The γ″ phase is the major strengthening phase of the GH3625 alloy, and usually distributes dispersedly. The γ″ phase with Ni3Nb stoichiometry, has lattice parameters of a = 0.362 nm and c = 0.740 nm. The formation of the δ phase slightly reduces the intergranular corrosion resistance. The δ phase precipitates when the samples age at above 700 °C and below 950 °C for a long time. If an aging temperature of 650 °C is selected, from the perspective of precipitation kinetics this temperature is exactly the optimal precipitation temperature of the γ″ phase. There are still two types of intermetallic phases in thermodynamic equilibrium, the P phase and the σ phase (Figure 1), which have similar compositions. The σ phase transforms into the P phase as the temperature drops below 720 °C. The σ phase, commonly harmful for corrosion-resistant alloys, is the topologically close packed (TCP) phase that theoretically exists in the GH3625 alloy. Its tendency to precipitate is relatively less if there is reasonable heat treatment of the solution. The P phase only exists theoretically in thermodynamic calculations and cannot meet its kinetic precipitation conditions in practice.

**Figure 3:**
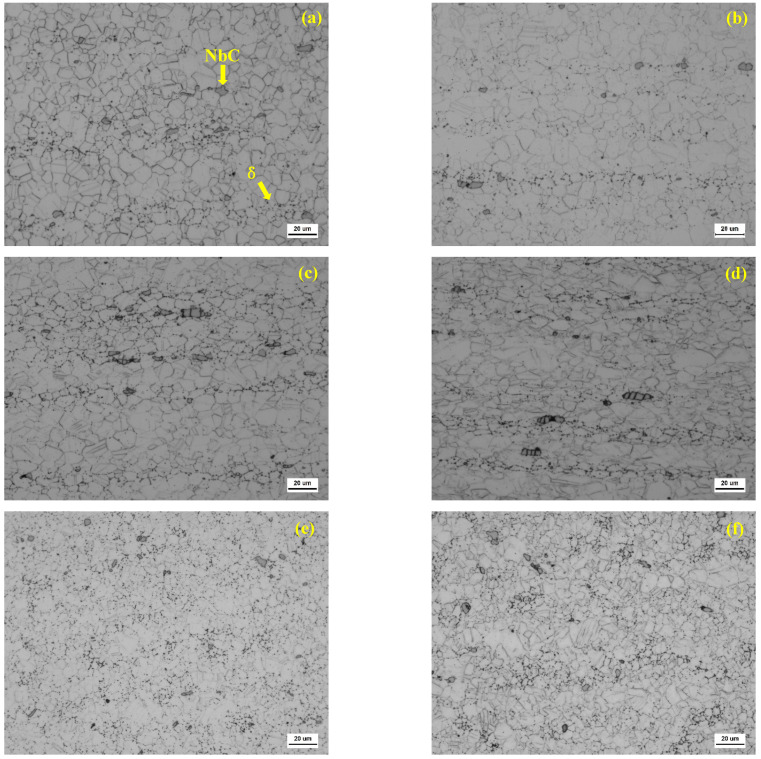
OM microstructure of cold-drawn GH3625 alloy with different reductions in area (500×): (**a**) 0%, (**b**) 10%, (**c**) 20%, (**d**) 30%, (**e**) 30% + aging at 760 °C for 1 h, and (**f**) 30% + aging at 650 °C for 24 h.

Figure 3 shows the microstructure of the GH3625 alloy with different reductions in area. As seen, cold drawing has a relatively small effect on the precipitation of GH3625, slightly refining the grain size, and significantly changing the carbides. As shown in Figure 3a, the grains without cold drawing are mostly equiaxed, and the overall microstructure retains the flow left by the hot deformation process, and the carbide is nodular. As shown in Figure 3b, when the area decreases by 10%, the carbides have a slight tendency to crack, but the overall microstructure changes little. Figure 3c shows that when the reduction in area increases to 20%, great tensile cracking (above 10 µm) occurs in the large-sized carbides, and the number of twins increases. Figure 3d shows that when the reduction in area increases to 30%, slightly smaller carbides (3–10 µm) are also broken, and multiple grains are elongated, resulting in an overall increase in grain size by 1 level (ASTM). As Figure 3e,f show, different aging treatments were performed on the samples with a 30% reduction in area after cold drawing, and was found that the precipitates at the grain boundaries only slightly increased. This is because the aging at 760 °C for 1 h makes the precipitates change slightly: M23C6 does not precipitate, while aging at 650 °C for 24 h separates out some of the γphase, which is so small that it cannot be observed by optical microscope.

**Figure 4:**
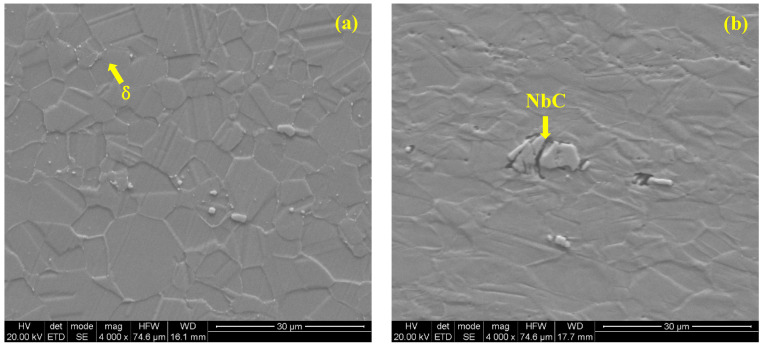
SEM microstructure of cold-drawn GH3625 alloy with different reductions in area (4000×): (**a**) 0%, (**b**) 30%.

Figure 4 shows the change in microstructure caused by cold deformation can be more clearly demonstrated by SEM, as described above. And it also clearly shows that the precipitates of secondary carbide, shown as spots, are almost entirely at grain boundaries, distributed parallel to the direction of hot work along the segregation bands, which is almost unrelated to cold work.

**Figure 5:**
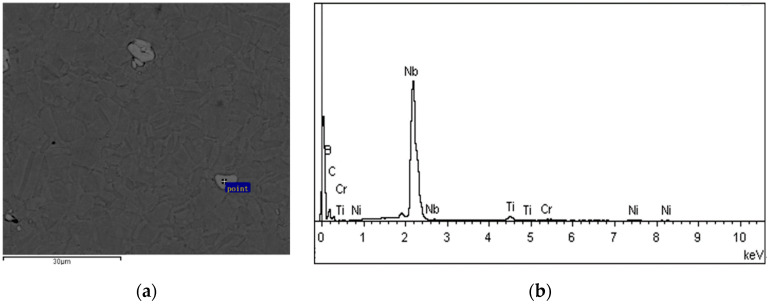
Carbide EDS of cold-drawn GH 3625 alloy: (**a**) back-scattered electron (BSE) image of carbides, (**b**) EDS spectrum of selected points.

The composition of precipitates of the samples after cold drawing with a 30% reduction in area and aging at 650 °C for 24 h was analyzed by energy dispersive X-ray spectroscopy (EDS). As shown in Figure 5 and Table 3, the precipitates are mainly NbC, and the semi-quantitative analysis shows a 61.5% mass fraction of Nb. The only visible precipitates in the structure are NbC, because the holding temperature of 760 °C is low and the holding time is short. In addition, the low carbon content results in no precipitation of the M23C6 phase. Theoretically, the γ″ phase should be precipitated in the microstructure after aging for 24 h, but it is small to nanoscale, making it difficult to characterize.

**Figure 6:**
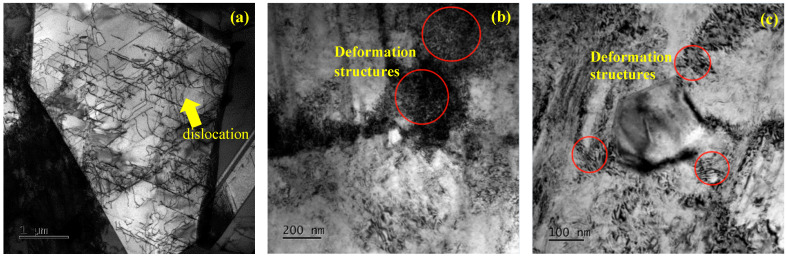
Bright-field images (TEM) of the microstructure before and after cold drawing. (**a**) Dislocation microstructure within grains and at grain boundaries before cold drawing, and the g vector indicates the dislocations within the grains before cold drawing. (**b**) Deformation of dislocation pile-up at grain boundary for the sample with 30% reduction in area. (**c**) Deformation of dislocation pile-up near second phase for the sample with 30% reduction in area.

Figure 6 shows the bright-field images (by TEM) of the microstructure before and after cold drawing. Figure 6a shows that there are some dislocation lines within the grains, and the distribution of the dislocations at the grain boundaries is denser. The grain boundaries may be the origin of dislocations. This is because the experimental bar was initially hot-rolled from alloy ingots, and although heat treatment was carried out after rolling, it was not enough to completely eliminate all dislocations in the structure. Therefore, there were some dislocations before cold drawing. As shown in Figure 6b,c, a large number of dislocation lines occur in the microstructure of the samples. Figure 6b shows a lot of dislocation pile-up and tangling at grain boundaries, while Figure 6c shows the dislocation pile-up near the second phase. As can be seen from the above, the numerous dislocation pile-ups at grain and phase boundaries lead to the deformation structures.

**Figure 7:**
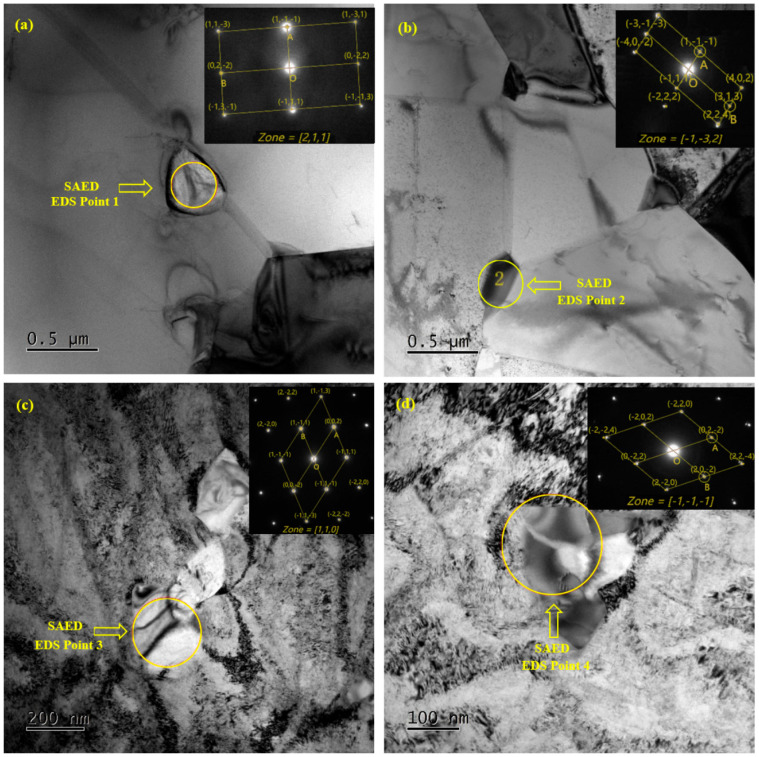
Images (TEM) before and after cold drawing. (**a**) Bright-field image with 0% reduction in area, and corresponding SAED of MC phase; (**b**) bright-field image and corresponding SAED of M6C phase before cold drawing; (**c**) bright-field image with 30% reduction in area, and corresponding SAED of MC phase after cold drawing; (**d**) bright-field image with 30% reduction in area, and corresponding SAED of M23C6 phase after cold drawing.

Figure 7 shows the selected area electron diffraction patterns (SAED) of the precipitate phases. Combined with EDS analysis (Table 4), it can be found that the main precipitates are in the NbC phase. Comparing Figure 7a,b with Figure 7c,d, it can be seen that there is a small change in the types of precipitates before and after drawing, mainly in MC-type carbides. In the sample without cold drawing, the MC phase (in Figure 7a) and a small amount of the M6C phase (in Figure 7b) are found. In the cold-drawn sample, the MC phase (in Figure 7c) and M6C phase are found, as well as a small amount of the M23C6 phase (in Figure 7d).

**Figure 8:**
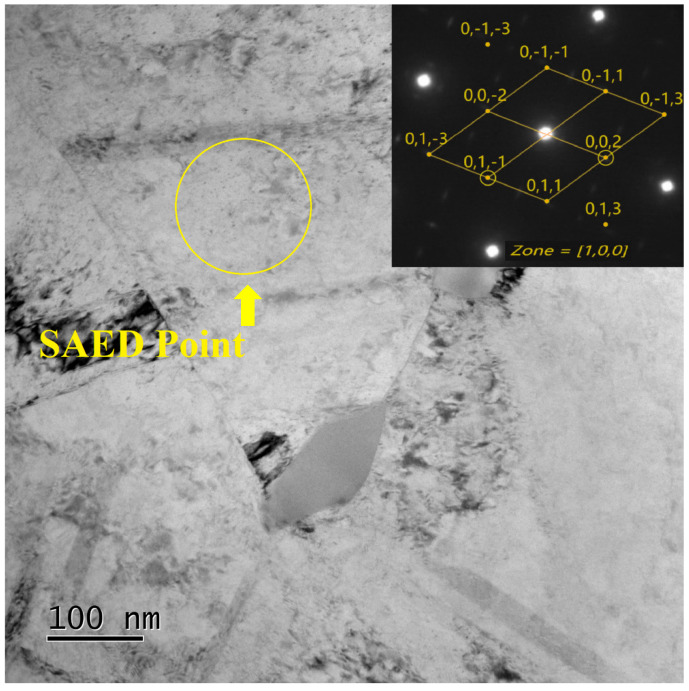
Morphology and SAED image of γ″ phases.

A small number of dispersed and tiny γ″ phases are observed in the microstructure of the cold-drawn samples after aging at 650 °C for 24 h, which has some consistency with the results displayed by the dynamic curve. The morphology is shown in Figure 8.

### 3.2. Tensile Properties

**Figure 9:**
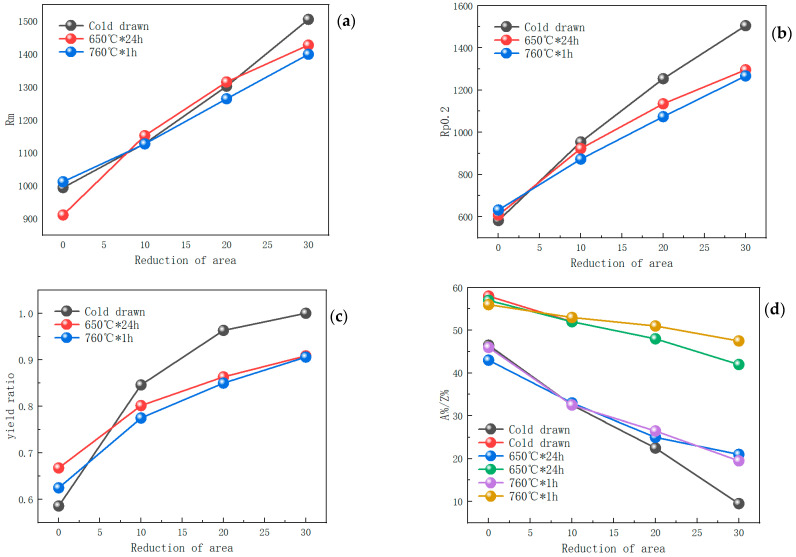
Tensile properties of cold-drawn bars with different reductions in area at room temperature. (**a**) Tensile strength, (**b**) yield strength, (**c**) yield ratio, and (**d**) elongation and reduction in area.

The tensile properties of samples with different deformation and heat treatment states were tested at room temperature. The yield strength (Rp0.2), tensile strength (Rm), elongation (A%), and section shrinkage (Z%) are shown in Table 5 and Figure 9.

Figure 9a shows that cold deformation significantly improves the strength, with a strength increase of 130–200 MPa for every 10% increase in reduction in area. Heat treatment has little effect on the tensile strength of the samples with ≤20% reduction in area. For the sample with a 30% reduction in area, recovery occurs following heat treatment, resulting in a decrease in strength of about 100 MPa. Figure 9b shows that the strength increase caused by cold drawing is more pronounced in the yield strength, which increases by 250–370 MPa for every 10% increase in reduction in area. Meanwhile, aging can slightly enhance the yield strength of the undeformed sample by the second phase, and its strengthening mechanism is based on the formula for the second-phase strength increase [30].

$$\tau =0.8*M\frac{Gb}{\lambda }$$

τ is the strength increase, *M* is the Taylor factor (=3), *G* is the shear modulus, *b* is the Burgess vector, and *λ* is the particle spacing in the second phase.

Heat treatment has a little effect on the mechanical properties of the alloy with 10% strain, while it has a greater impact on the alloys with 20% and 30% deformation. This is because during the cold drawing process, when the strain is small (10%), the strengthening effect from the edge to the center decreases, so the strengthening effect in the center is weak. However, the tensile samples could only be chosen from the center of the bar. Therefore, the heat treatment has little effect on the core of the sample with a 10% reduction in area. Meanwhile, it can be seen that the yield strength of the sample held at 650 °C is higher than that of the sample held at 760 °C for 1 h. This is because the γ″ phase starts nucleation at 650 °C, and although it has not yet grown due to the relatively short time, it will strengthen the second phase from a microscopic perspective. At 760 °C, there are fewer precipitated secondary carbides, and the main carbides in the microstructure are still the large primary carbides during solidification, which have little effect on improving strength. Therefore, the smelting temperature should be properly controlled, which could reduce the number of primary carbides and make the carbides precipitate in the form of small secondary carbides, thus benefiting the performance of the alloy. Figure 9c shows that as the reduction in area increases, the yield strength ratio approaches 1, which is very unfavorable for the safe service of the material. When the yield strength ratio is appropriately reduced and the stress is greater than the yield strength, the material will undergo plastic deformation in advance, which will make the failure signal more obvious, providing reaction time for component replacement and reducing the losses caused by material failure in the entire system. Both heat treatment processes can significantly reduce the yield ratio from 1 to around 0.9. Figure 9d shows that the plasticity significantly decreases as the reduction in area increases. Meanwhile, the two heat treatment processes improve the elongation with 30% strain, which is attributed to the recovery effect. However, the change in dislocation does not damage the grain boundaries. After necking occurs, the alloy can still continue necking, which also makes the influence of heat treatment on the reduction in area small.

**Figure 10:**
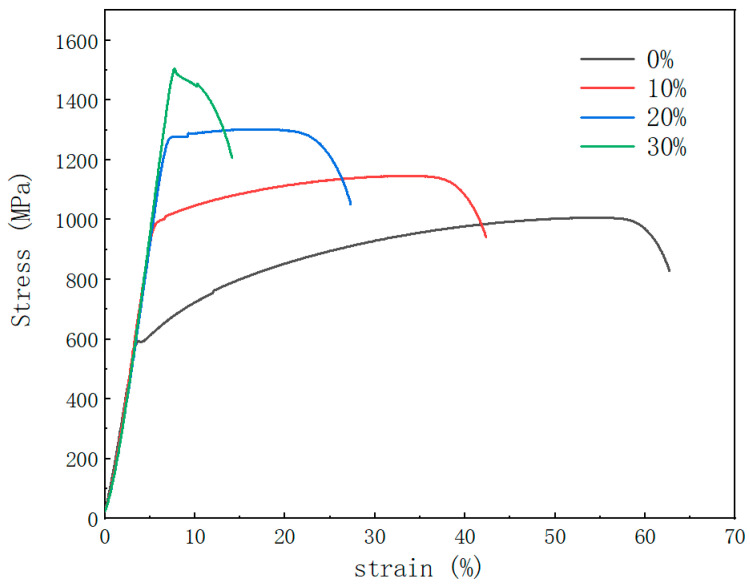
Stress–strain curves with different reductions in area.

The stress–strain curve reflects the deformation behavior of the material at various stages. Figure 10 shows that the curves of the elastic stage almost overlap for samples with different reductions in area, but there is an obvious difference in the plastic strain stage. The two main factors affecting the plasticity index are the plastic uniform extension after elastic strain, and the effect of necking after exceeding the maximum stress. In this experiment, the necking of all samples was similar, so the elongation was significantly affected by the former factor. The main factors determining the plastic deformation ability of the alloy in the uniformly elongation section are its dislocation movement and work-hardening ability. When the sample is subjected to tensile stress load, the parallel segments gradually become thinner, then a large number of dislocations occur. The first part that becomes thinner bears the maximum stress, but due to the large increase in the number of dislocations and their movement, work hardening occurs, which enables the alloy to have a self-healing mechanism. After local strengthening, the fracture ultimate strength at this point increases, and continues to extend uniformly until fracture. The premise of this experiment is cold drawing, which has led to the formation of a large number of dislocations in advance. The pile-up of a large number of dislocations obviously hinders the further increase and movement of dislocations, slowing down the production of new dislocations and their movement to the weakest position in the cold-deformed sample, reducing its ability to uniformly extend, and thus, reducing the overall elongation of the material after forging.

**Figure 11:**
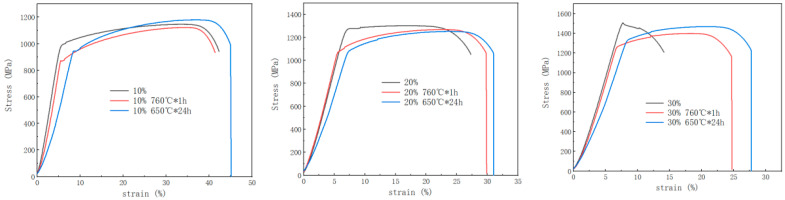
The influence of heat treatment on the mechanical properties with various reductions in area.

Figure 11 shows the influence of heat treatment on the stress–strain curve, which is mainly reflected in samples with large reductions in area. Both heat treatment processes can restore the stress–strain curve of a sample that has been strengthened by a large number of dislocations to the shape of the stress–strain curve before cold drawing. The only difference is that the turning point (yield strength) improves. It can also be seen that the sample aged at 650 °C has a larger elastic strain under the same load.

The changes in the mechanical properties of GH3625 after cold drawing were studied. Ding et al. studied the relationship between cold deformation and the mechanical properties of GH3625. It was found that cold deformation was the main factor affecting work hardening [31]. Zhao studied the effect of different degrees of cold deformation on the mechanical properties. It was found that when the cold rolling deformation was 20%, the tensile strength could reach 1050 MPa. With an increase in reductions in area, the strength increased. The condition of the material was different from that in this study, so the experimental data obtained are not the same. However, the overall trend and strengthening mechanism of the materials obtained from the research are similar to the direction of this study [19].

### 3.3. Hardness Gradient

**Figure 12:**
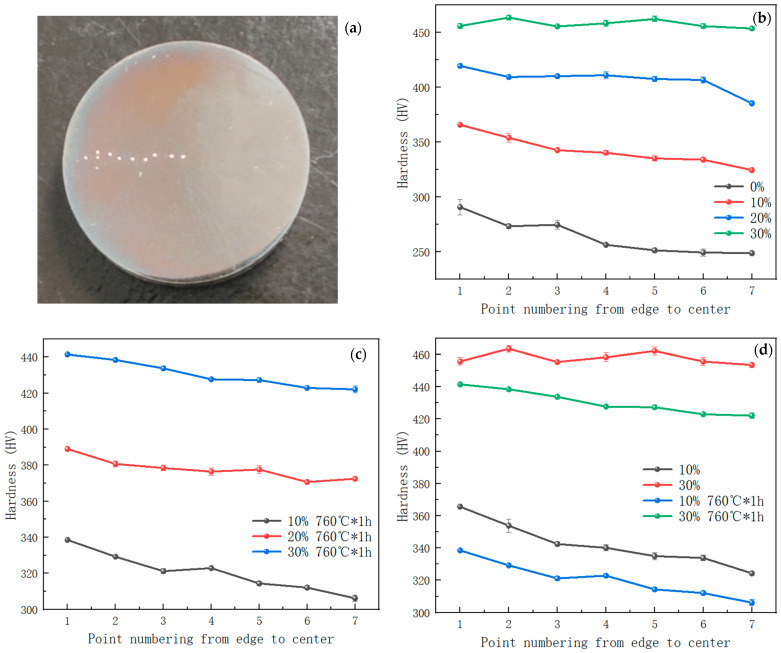
Hardness after cold drawing and heat treatment. (**a**) Macroscopic photo; (**b**) hardness with different reductions in area; (**c**) hardness after aging at 760 °C; and (**d**) hardness before and after aging treatment with 10% and 30% reductions in area.

The Vickers hardness of samples before cold drawing, after cold drawing, and after heat treatment were tested from the edge to the center. Figure 12 shows that due to the annealing following the hot rolling, the hardness tested from the center to the edge of the steel bar is uniform before cold drawing. When the reduction in area is 10%, the strengthening effect of the edge of the cold-drawn steel bar is stronger, and the hardness values from the edge to the center decrease monotonically, with a difference of 41 HV between the edge and the center. When the reduction in area is 20%, the hardness of the steel bar after cold drawing increases, but the deformation still cannot be fully transmitted to the center. When the reduction in area is 30%, the strength of the steel bar after cold drawing is uniform, and the hardness values at the center and edges are close. This indicates that when the total cold deformation is small, the strain at the edges of the steel bar is larger and the strain at the center is smaller. As the total deformation increases, the strain at the center increases, approaching that at the edge.

The hardness of samples after heat treatment was tested. For samples aged at 760 °C for 1 h with 10% and 30% reductions in area, it is found that heat treatment cannot completely eliminate the work hardening influence. The hardness value at different positions of the sample after heat treatment decreases by about 30 HV, and the hardness value from the edge to the center of the sample still keep decreasing, which is consistent with that of the sample after cold drawing without heat treatment.

### 3.4. Dislocation Density

**Figure 13:**
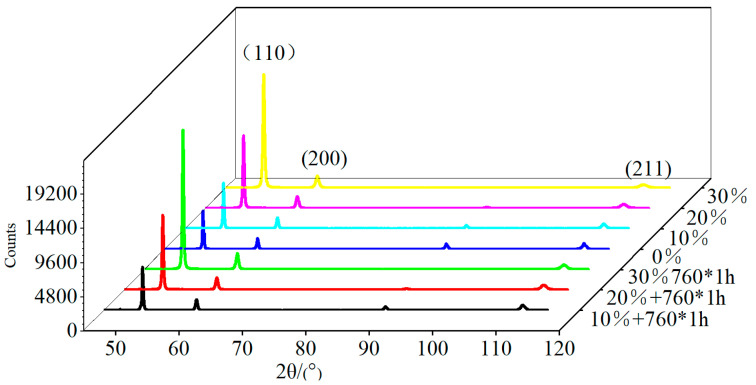
XRD map of samples with different reductions in area.

There are usually two methods for calculating dislocation density. One is to use TEM for microscopic statistics, which uses the ratio of the total length of all dislocation lines per unit field of view to the area to represent the facial density of dislocations. This method is inaccurate and it can only indicate the facial density of dislocations in a certain microscopic area, with units of nm^−1^. Another method is to use XRD to test and calculate the average dislocation density. The test results are shown in Figure 13.

Firstly, the average thickness of the crystallites perpendicular to the crystal face is calculated, then the D value calculated by different 2 θ angles is averaged, and finally, the volume density of dislocations δ is calculated, with units of cm^−2^. This is calculated by the Scherer equation [32,33]:

$$D=\frac{k\lambda }{\beta cos\theta }$$

where *D* is the crystallite size, *k* is the Scherrer constant (=0.89) [34], β is the measured width at half maximum of the diffraction peak of the sample, θ is the Bragg diffraction angle, λ is the wavelength of the X-ray, Co target, with a wavelength of 1.7889 Å.

$$\delta =\frac{1}{D^{2}}$$

Then, the equation above can be used to calculate the dislocation density *δ* [35]. Test results of the dislocation density with different reductions in area and aged at 760 °C for 1 h are shown in Table 6.

It can be seen in the table that a 10% reduction in area increases the dislocation density by 8 times, and for every 10% increase in reduction in area thereafter, the dislocation density increases by 3 times. After heat treatment, the dislocation density significantly decreases, and the larger the reduction in area, the greater the decrease in dislocation density. The dislocation density of the sample with a 10% reduction in area decreases to 72%. The dislocation density of the sample with a 20% reduction in area decreases to 50%. The dislocation density of the sample with a 30% reduction in area decreases to 25%. This is related to the heat treatment temperature, where 760 °C is much higher than the temperature at which the material is recover. During the heat treatment process, a large number of dislocations ablate, and the lattice distortion energy is fully released due to static recovery at this temperature.

Deformation strengthening is one of the main methods to improve the strength of material. The yield strength values strengthened by four different strengthening methods are the superposition of effects caused by the different methods. When there is fine-grained strengthening and dislocation strengthening, the two are superimposed using the sum of square roots [36,37]. In this experiment, solution strengthening and the change in grain size are not obvious. The strength increase brought about by the second-phase strengthening is estimated to be 50 MPa based on the strength increase in the annealed and undeformed samples before and after aging, mainly calculating the contribution of dislocation strengthening.

The contribution of dislocations to the strength increase before and after cold drawing and heat treatment is calculated by the following formula [38,39]:

$$\sigma_{\rho }=aGb(\rho^{1/2})$$

where $\sigma_{\rho }$ is the strength increase; *α* is a constant, taken as 0.88; *G* is the shear modulus, taken as 79 GPa; *b* is the Burgess vector, taken as 0.25 nm; and ρ is the dislocation density. Table 7, Table 8 and Table 9 show the calculation results of samples with different dislocation densities.

Table 7, Table 8 and Table 9 show that the yield strength of the sample without cold drawing increases by 50 MPa after aging at 760 °C. It is believed that this strength increase is mainly caused by the strengthening of the second-phase precipitation. The increase in dislocation strength of the sample without cold deformation is 183 MPa, while the measured strength is 582 MPa. Therefore, the basic strength is considered to be 399 MPa in the calculations.

The calculation results show that the strength increase caused by 10–20% reductions in area and the strength reduction after heat treatment have a close relationship with the measured values. Although the specific theoretical values are slightly different from the measured values, the trend is very close. The theoretical dislocation strengthening of the material with a 30% reduction in area is enhanced sharply, with the yield strength reaching 2057 MPa, while the measured ultimate strength is only 1505MPa, and the yield ratio is 1 at this time. This is because materials with a yield ratio greater than or equal to 1 have extremely low plasticity, and the tested yield strength is forced to decrease. The material does not reach its yield strength during the tensile process, and due to the influence of ultimate strength, it fractures prematurely before yielding, resulting in a decrease in material safety. In summary, the good consistency between the calculated values and the measured values confirms that the strength increase is mainly provided by dislocation strengthening, reflecting the role of heat treatment in adjusting the mechanical properties of materials.

## 4. Conclusions

(1) The cold drawing process with 10–30% reductions in area can significantly improve the tensile strength and yield strength of the GH3625 alloy. After aging treatment at 650 °C and 760 °C, the yield strength decreased and the yield strength ratio greatly decreased. This study can provide more mechanical performance options for GH3625 alloy structural components in the petroleum and petrochemical industry.

(2) Proper heat treatment processes can ablate the residual dislocations after cold deformation and reduce the dislocation density in the microstructure of the GH3625 alloy, which is the main factor in reducing yield strength. It is clarified that the main strengthening mechanism in this study is dislocation strengthening by testing and calculating dislocation density under different states and the resulting strength increase. The precipitation strengthening effect caused by the second-phase precipitate during heat treatment provides an additional strength increase. Affected by γ″, the yield strength after aging at 650 °C for 24 h is higher than that after aging at 760 °C for 1 h. If further aging research can be carried out to study the trend in dislocation density changes after long-term holding, and to clarify and quantify the kinetics of the γ″ phase, the practical significance of this research work will be further deepened.

(3) The work-hardening gradient of cold-drawn material varies greatly with different reductions in area. When the deformation is small (10%), the gradient is obvious. When the deformation increases to 30%, the material is uniformly strengthened as the deformation is transmitted to the axis. Therefore, the actual strength provided by the workpieces with low reductions in area will be higher than that of the sample.

## 5. Prospects

This paper conducted a study on the mechanical properties of cold-drawn materials with 10–30% reductions in area and under aging at 650 °C and 760 °C. The research on cold deformation with a smaller reduction in area (3–10%) is not sufficient yet, so the effect of small cold deformation on surface strengthening should be further studied. In addition, the precipitation kinetics and mechanical performance after long-term holding at 650 °C have not been fully studied, and research on multi-step heat treatments at different temperatures is not sufficient. Afterwards, further adjustments can be made to the heat treatment process to provide more mechanical performance options.

## Figures and Tables

**Table 1:** Main chemical composition of niobium chromium master alloys (wt%).

Nb	C	S	Al	Si	Fe	Cr
23.65	0.05	0.001	0.11	0.10	0.23	Bal

**Table 2:** Main chemical composition of GH3625 (wt%).

C	Cr	Mo	Al	Ti	Nb	Fe	Ni
0.04	21.36	9.09	0.24	0.25	3.48	0.45	Bal

**Table 3:** Semi-quantitative chemical composition of carbides.

Element	Wt%	At%
Nb	61.5	17.5
C	25.1	55.4
B	10.4	25.6
Ti	1.3	0.7
Cr	0.6	0.3
Ni	1.1	0.5

**Table 4:** EDS analysis results.

Element	Point 1		Point 2		Point 3		Point 4	
	At%	Wt%	At%	Wt%	At%	Wt%	At%	Wt%
Nb	86.2	91.6	9.8	11.6	66.6	76.2	0.6	0.9
Ni	5.1	3.4	31.9	24.5	18.7	13.5	22.8	22.3
Cr	1.8	1.1	14.5	9.9	6.2	4	60.3	52
Fe	1.7	1	1.9	1.4	2	1.4	0.7	0.6
Ti	5.2	2.9	-	-	4.6	2.7	-	-
Mo	-	-	41.9	52.6	1.8	2.2	15.1	24
Al	-	-	-	-	-	-	0.5	0.2

**Table 5:** Tensile properties before and after cold drawing and heat treatment.

State	Reduction in Area	Rp0.2	Rm	A%	Z%
Cold drawn	0	582	994	46.5	58
	10	954	1128	32.5	52
	20	1254	1302	22.5	48
	30	1505	1505	9.5	42
650 °C for 24 h	0	608	911	43	57
	10	923	1152	33	52
	20	1135	1315	25	48
	30	1296	1427	21	42
760 °C for 1 h	0	632	1012	46	56
	10	873	1127	32.5	53
	20	1074	1264	26.5	51
	30	1267	1399	19.5	47.5

**Table 6:** Dislocation density in different states.

Reduction in Area	Cold Drawn	After Heat Treatment
0	1.1201 × 10^10^	
10	8.6161 × 10^10^	6.2325 × 10^10^
20	2.9209 × 10^11^	1.4214 × 10^11^
30	9.1703 × 10^11^	2.3126 × 10^11^

**Table 7:** Strength increase.

Reduction in Area	Cold Drawn/MPa	Aging at 760 °C/MPa
0	183	
10	508	432
20	936	701
30	1658	918

**Table 8:** Calculated yield strength.

Reduction in Area	Cold Drawn/MPa	Aging at 760 °C/MPa
0	582	
10	907	831
20	1335	1100
30	2057	1317

**Table 9:** Measured yield strength.

Reduction in Area	Cold Drawn/MPa	Aging at 760 °C/MPa
0	582	632
10	954	873
20	1254	1074
30	1505	1267

## Data Availability

Data are contained within the article.
